# Sensitive Detection of Single-Nucleotide Polymorphisms by Solid Nanopores Integrated With DNA Probed Nanoparticles

**DOI:** 10.3389/fbioe.2021.690747

**Published:** 2021-06-30

**Authors:** Ling Zhi Wu, Yuan Ye, Zhi Xuan Wang, Die Ma, Li Li, Guo Hao Xi, Bi Qing Bao, Li Xing Weng

**Affiliations:** ^1^Key Laboratory for Organic Electronics and Information Displays, Jiangsu Key Laboratory for Biosensors, Institute of Advanced Materials, National Synergetic Innovation Center for Advanced Materials, Nanjing University of Posts and Telecommunications, Nanjing, China; ^2^College of Geography and Biological Information, Nanjing University of Posts and Telecommunications, Nanjing, China

**Keywords:** single nucleotide polymorphism, gold nanoparticle, gastric cancer, solid nanopore, DNA probe

## Abstract

Single-nucleotide polymorphisms (SNPs) are the abundant forms of genetic variations, which are closely associated with serious genetic and inherited diseases, even cancers. Here, a novel SNP detection assay has been developed for single-nucleotide discrimination by nanopore sensing platform with DNA probed Au nanoparticles as transport carriers. The SNP of p53 gene mutation in gastric cancer has been successfully detected in the femtomolar concentration by nanopore sensing. The robust biosensing strategy offers a way for solid nanopore sensors integrated with varied nanoparticles to achieve single-nucleotide distinction with high sensitivity and spatial resolution, which promises tremendous potential applications of nanopore sensing for early diagnosis and disease prevention in the near future.

## Introduction

Single-nucleotide polymorphisms (SNPs) primarily refer to single-nucleotide substitution that constitutes the most common genetic variation, with an average occurrence of ∼1/1,000 base pairs, which are closely associated with various cancers and tumors (Liu et al., 2017; Varona and Anderson, 2019; Megalathan et al., 2021). As an important biomarker, numerous methods have been developed for detecting SNPs. The conventional approaches are polymerase chain reaction amplification and sequencing, which are adequate to know each gene site of DNA fragment, but overqualified with being time-consuming and costly (Halperin and Stephan, 2009; Maguin and Marraffini, 2021). A variety of biosensors have been performed involving fluorescent labeling, chemiluminescence, and microassays (Liu et al., 2017; Tian et al., 2018; Zhou et al., 2020; Zhang et al., 2021). However, the photoelectric signals obtained from these methodologies have relatively low specificity and false-positive probability for single-nucleotide discrimination in pathogenic mutants. Hence, novel SNP assays with respect to simplicity, sensitivity, high throughput, and low cost are still in demand for the earlier diagnosis and clinical prognostics.

As an emerging single-molecule detection technology, nanopores are promising sensors for DNA identification, proteomic detection, determination of epigenetic changes, and biomolecular mechanism exploration with the advantages of being label-free and high-throughput (Wang et al., 2017; Jeong et al., 2019; Spitzberg et al., 2019; Chen et al., 2021; Fragasso et al., 2021; Hu et al., 2021). The nanopore technique is inspired by the transmembrane protein channels embedded in lipid bilayers that allow ions and molecules inside and outside the living cell to exchange freely. Based on the pore materials, nanopores have been developed into two major types of biological and solid-state pores, and both take their respective advantages to achieve single-molecule identification toward clinical detection (Wang et al., 2017, 2018, 2020; Meng et al., 2019). However, the spatial and temporal resolution of individual nucleotides discriminated by nucleotide-specific current signals remains a challenge for nanopore sensing. For instance, the natural fixed pore size and instability of biological nanopores limit their sensing application. For solid nanopores with mechanical robustness and size controllability, the accuracy and the limited bandwidth of the current measurement systems are technical hurdles at single-nucleotide resolution. In this study, a novel SNP assay system has been proposed based on the nanopore readout platform integrated with DNA-probed gold nanoparticles. The functionalized Au nanoparticle is suitable for a good transport model for nanopore sensing. For instance, Au nanoparticles with a certain volume can produce distinct ionic current signatures through nanopores. Moreover, nanoparticles loaded with DNA probes facilitate molecular translocation to improve accuracy and the signal-to-noise ratio. The DNA probes wrapped on Au nanoparticles favor hybridization with SNP sequences with a high selectivity for single-nucleotide discrimination (Ang and Lanry Yung, 2012; Venta et al., 2013, 2014; Karmi et al., 2021). The perfectly matched hybridization of SNP sequences with DNA probes absorbed on Au nanoparticles will trigger the nanoparticle assembly to form dimers. The distinction of SNP mutations can be easily achieved by observing the differences of signals between the monomers and dimers of nanoparticles translocated through the hole by a nanopore platform. Thus, with high selectivity, efficiency, and simplicity, this method can be used to successfully distinguish single-nucleotide variations of DNA targets independent of the nanopore morphology.

## Materials and Methods

### Materials

The chemicals used in this experiment were obtained from commercial sources: trisodium citrate dihydrate (Fisher), HAuCl_4_.3H_2_O (Sigma), KCl (Sigma-Aldrich), concentrated sulfuric acid (Sinopharm Chemical Reagent Co., Ltd.), aquaehydrogenii dioxide (Sinopharm Chemical Reagent Co., Ltd.), and bis(p-sulfonatophenyl)phenylphosphine dihydrate dipotassium salt (BSPP) (Sigma-Aldrich, St Louis, MO, United States). All DNA oligonucleotides were synthesized by Shanghai Sangon Biotechnology Co., Ltd. (Shanghai, China). The sequences of DNA probes are PolyA-1 (60AGCGGACTCCAACACTCCGT) and PolyA-2 (60ACTGCCCATGGTGGGGGCAG), respectively. The wild target (P53WT) of the p53 gene is GAGGTTGTGAGGCGCTGCCCCCACCATG, and that of SNP targets (P53MU) is GAGGTTGTGAGGC ACTGCCCCCACCATG. Au nanoparticles were purchased from Ted Pella (Redding, CA, United States). Milli-Q super-purified water with a resistance >18 MΩ/cm was used in all the experiments.

### Preparation of DNA Modified Au Nanoparticles

The Au nanoparticles were firstly protected using phosphine moiety bis(p-sulfonatophenyl) phenylphosphine to increase their stability, as previously described (Zhu et al., 2016; Chen et al., 2017). These phosphine-coated Au nanoparticles were incubated with diblock DNA with a 1:10 ratio for 16 h with gently shaking at room temperature. Then, 1 M of sodium phosphate buffer (1 M of NaCl, 100 mM of Na_2_HPO_4_, and NaH_2_PO_4_, pH 7.4) was added into the DNA/Au nanoparticle mixture for five times with a 30-min interval to reach a final concentration of 100 mM NaCl. This mixture was incubated for 24 h at room temperature. Next, the resulting mixture was centrifuged at 12,000 rpm for 20 min to remove excess DNA. The nanoparticles were resuspended in 0.1 M sodium phosphate buffer (PBS, 0.1 M of NaCl, 10 mM of Na_2_HPO_4_, and NaH_2_PO_4_, pH 7.4). The DNA-functioned Au nanoparticles were also characterized by scanning electron microscope (SEM) images from Axiostar Plus (Zeiss Axiostar Plus).

### Nanopore Fabrication and Data Acquisition

The nanopores used in all our experiments were fabricated using focused-ion-beam drilling with Ga^+^ ions. The resulting pores were visualized through SEM Axiostar plus (ZEISS Axiostar plus). Chips were cleaned in piranha solution (3:1 v/v H_2_SO_4_:H_2_O_2_) at 80°C for 30 min in order to remove organic contaminants and to facilitate pore wetting. The prepared nanopore chips were then sealed into polydimethylsiloxane (PDMS) microfluidic channels. The Ag/AgCl electrodes were put in each chamber to connect to a pico-Ampere current amplifier Axopatch 700B (Molecular Devices), which applied a transmembrane voltage and recorded the nanopore ion current.

## Results and Discussion

### Design of DNA Probe Wrapped on Au Nanoparticles

**FIGURE 1:**
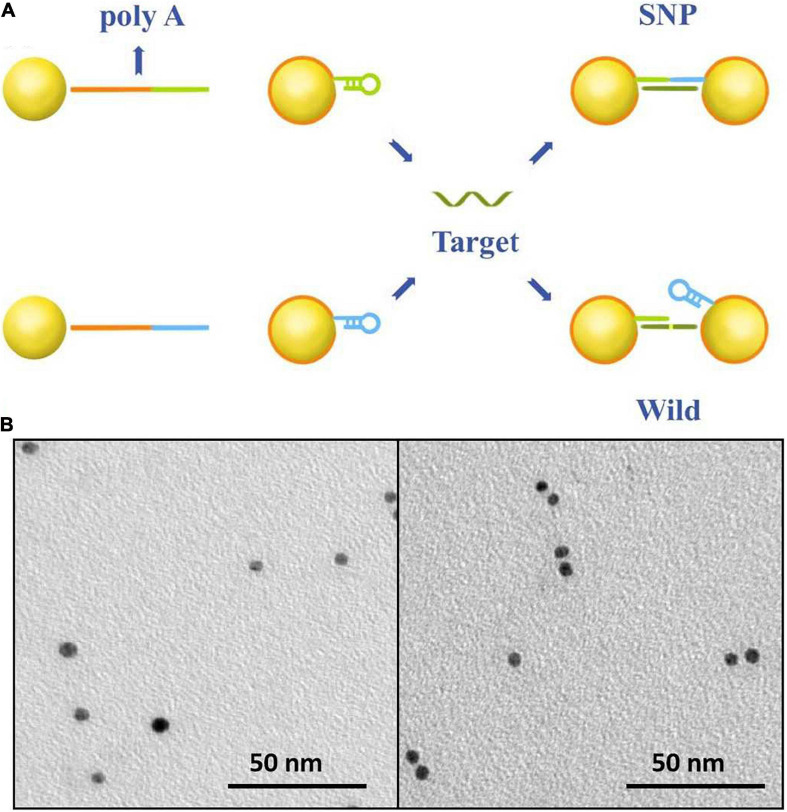
**(A)** The work principle of the SNP detection system. The Au nanoparticles firstly were labeled by blocking DNA strands with the poly-adenine (polyA) block (red) and the recognition (blue and green). One block of polyA oligonucleotide could strongly absorb on the surface of gold nanoparticles; the other was designed to perfectly complementarily hybridize with SNP targets and mismatch to the wild-type targets. **(B)** SEM images of DNA-probed Au nanoparticles with wild targets in the form of monomer and SNP targets in the form of dimer.

The sensing principle of single-nucleotide discrimination based on the nanopore platform is illustrated in Figure 1A. A diblock DNA probe has been designed to perfectly match with SNP targets. The probe consists of a binding chain of polyA sequences and a capture chain of complementary sequences. The polyA block naturally adheres to the gold surface via adenine adsorption. By rational design of the polyA length, the gold nanoparticles are wrapped by DNA probes at single-molecular level, and the appended recognition blocks with an upright conformation favor DNA recognition (Qin and Lanry Yung, 2007; Yao et al., 2015; Zhu et al., 2016; Chen et al., 2017). The recognition strands in blue and green are perfectly complementary to SNP targets and spontaneously form hairpin structures to reduce non-specific adsorption between nucleotide sequences. After addition of SNP targets, DNA probes immobilized on the nanoparticles preferentially capture SNP targets by the perfectly matched sequence hybridization. A set of discrete Au-DNA conjugate dimers have been formed via DNA-hybridizing fragments as linkers. The wild-type target with a mismatched base is hard to open the hairpin structure and initiates self-assembly of Au nanoparticles. Thus, the Au-DNA probe has a high selectivity to discriminate the SNP and wild targets.

The recognition of the Au-DNA probes and targets is verified in Figure 1B. It is clear that Au nanoparticles are dispersed as monomers in the aqueous solution prior to the addition of wild-type and mutation targets. Once the SNP targets are added, DNA probes anchored on the Au particles are opened and perfectly hybridized with SNP sequences to link two Au spheres into a dimer, while the recognition of the DNA probe is hard to initiate hybridization reaction owing to sequence mismatch with wild targets. The results are further verified in light of the scattering spectra. The diameter of Au nanoparticles in mixture ranges from 5 to 10 nm after SNP targets hybridized with DNA probes. Hence, the designed DNA probes anchored on Au nanoparticles are suitable to capture SNP sequences and the discrete conjugate complex can be identified by the nanopore platform with a high selectivity for single-nucleotide discrimination.

### Discrimination of Wild and Mutant Targets in Nanopore Sensing

**FIGURE 2:**
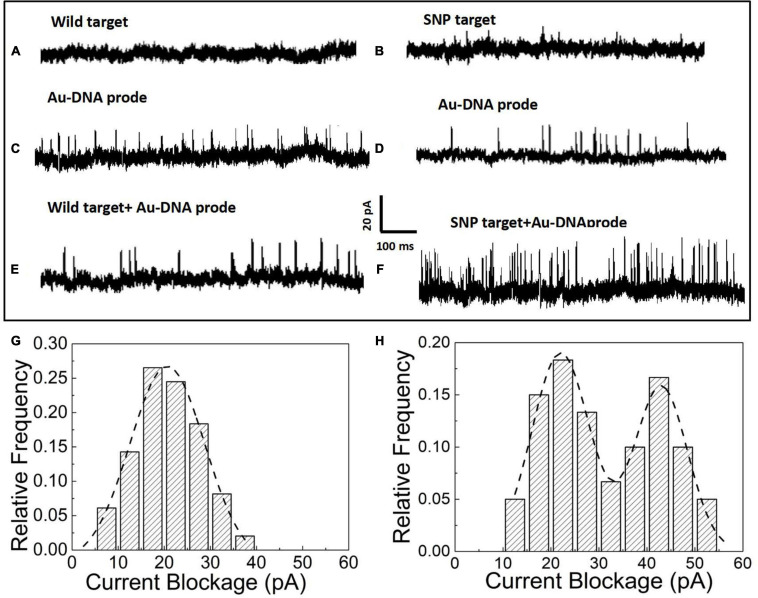
**(A)** Current signal in the presence of wild targets. **(B)** Current signal in the presence of SNP targets. **(C)** Current signal in the presence of the Au-DNA probe. **(D)** Current signal in the presence of Au-DNA probes. **(E)** Current signal in the presence of wild targets with Au-DNA probes. **(F)** Current signal in the presence of SNP targets with Au-DNA probes. **(G)** Histograms of the blocked current amplitudes with wild targets. **(H)** Histograms of the blocked current amplitudes with SNP targets.

After the hybridization of DNA probes on the nanoparticles with wild and SNP targets, the nanoparticles in the form of monomers and dimers have been transported to the nanopore platform to pick up individual distinct signals for SNP detection. In the nanopore device, the prepared nanoparticle samples have been added into reservoirs and driven through an orifice separated by a silicon nitride membrane sandwiched between two Ag/AgCl electrodes. The ionic current flowing through the nanopores is momentarily interrupted as particles pass through the pore. Thus, a set of upward pulses are observed in the current–time traces shown in Figure 2. The nanopore with a diameter of 35 ± 3 nm is used to capture the Au particles into the pore and acquires the optimal current pulses with a high signal-to-noise ratio. In Figures 2A,B, as pure wild and SNP targets are loaded into the reservoirs, no blockage signals appear in the current trace since the short DNA fragments of a dozen nucleotide bases are too small to generate discernable signals in voltage clamp mode. However, the addition of Au nanoparticles with DNA probes triggers a series of current pulse signals, shown in Figures 2C,D. The blockage current is induced by Au nanoparticles flowing into the pore in the form of monomers. After addition of wild and SNP targets as shown in Figures 2E,F, the blocked current signals have been enhanced as SNP targets are hybridized with complementary DNA probes wrapped on the Au nanoparticles, while the current pulses in the presence of wild targets are still similar to that of Au-DNA probes.

The blockage signals from individual particle translocations can be distinguished by the time duration (t_*d*_) and the magnitude of the blockage current (I_*b*_). The histograms of the magnitude and dwell time of the translocation events have been statistically sorted to plot the columnar statistical graphs, as shown in Figures 2G,H. The amplitude distribution of blockage events is fitted by Gaussian models. Based on the fitting curves, the peak values of the blocked current are maximized at 20 ± 2 pA, which corresponds to the most probable current drops induced by monomer particles through the nanopore at biased voltages. Once the SNP targets are added and react with the DNA probes, an intriguing phenomenon of double peaks appear in the current blockage statistics histograms. One of the peaks at 19 ± 3 pA is clearly similar to the most probable amplitude intensity of the blocked current events induced by monomer nanoparticles. A larger peak at the current of 41 ± 5 pA appears, which is about twice as large as the first peak. Based on the volume exclusion theory of the particles entering the pore, the blockade signals are directly proportional to the particle volume as the following formula: $\Delta \,I_{b}\,\left(t\right)=-\frac{\sigma \,\phi }{H_{e\,f\,f}^{2}}\,\Lambda \,\left(t\right)\,\left[1+f\,\left(\frac{d_{m}}{D_{p}},\frac{l_{m}}{H_{e\,f\,f}}\right)\right]$, where *σ* is the solution conductivity, *ϕ* is the applied voltage between the electrodes, *Λ* is the excluded volume of a translocation particle inside the pore, *H*_*eff*_ is the effective length of the nanopore, *d*_*m*_ is the diameter, *l*_*m*_ is the length of a particle molecule, *D*_*p*_ is the nanopore diameter, and *f*(*d*_*m*_/*D*_*p*_,*l*_*m*_/*H*_*eff*_) is a correction factor (Talaga and Li, 2009). It is clear that Au nanoparticles are assembled into dimers by the hybridization between SNP and DNA probes. Thus, the smaller of the current peaks appears owing to monomer particle translocation, while the larger one arises from the assembled dimers passing into the pore.

### Quantification of the Targets

**FIGURE 3:**
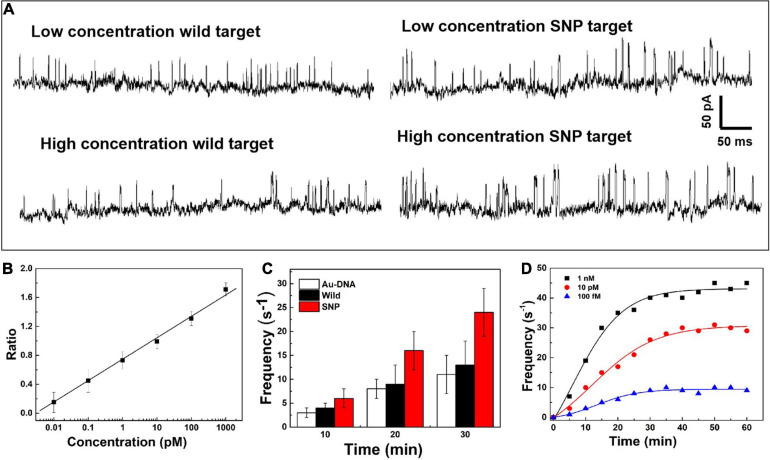
**(A)** Current signals in the presence of the SNP target and wild target at low and high concentrations. **(B)** Log plot of the capture ratio of the dimer and monomer obtained from blockage current versus SNP concentrations. **(C)** Signal frequency versus experimental time. **(D)** The capture frequency of blocking events dependent on time at different concentrations.

On the nanopore readout platform, single-nucleotide discrimination between SNP and wild targets has been detected by the signal amplification of the DNA-probed nanoparticles as transport carriers through the nanopore. Considering the high-sensitivity demand in the earlier stage of cancer and tumor diagnosis, the change of the current signals has been further explored dependent on the concentration of SNP targets, as shown in Figure 3A. The ionic current fluctuation is observed as the DNA-probed Au nanoparticles are added into the pore, and a set of spike-like signals appears due to the volume exclusion effect of gold spheres passing through the nanopore one by one. After addition of SNP and wild targets, the recorded current trace shows an obvious change at low and high concentrations of targets. For addition of wild targets, the current trace is similar to the previous signals caused by individual Au-DNA nanoparticles. There is no visible change of the blockage current recording even though the concentration of the wild targets is increasing. The binding block of DNA probes hardly unfold the hairpin structure owing to the base-pair mismatch with wild targets. In contrast, more spike-like current signals continuously appear as increasing SNP targets. It is further verified that the designed DNA probes have high selectivity for SNP targets. From the statistical histogram of a large number of current pulse signals, the blockage current events with greater intensity and longer duration are characterized as a larger Gaussian peak position. The capture ratio of the dimer and monomer obtained from the statistical histogram of blockage current has been log-plotted as a function of SNP target concentration, as shown in Figure 3B. There is a linear relationship with SNP concentration increasing. At high SNP concentration, the capture frequency of dimers is greater than that of monomers, which indicates that the dimers formed by Au nanoparticle self-assembly are superior in mixed aqueous solution. The capture ratio is gradually reduced when the probability of the dimer formation is reduced with decreasing SNP concentration. By virtue of the nanopore–nanoparticle integrated approach, the SNP of the p53 gene mutation in gastric cancer is detected as low as to femtomolar under the experimental conditions. The sensitive detection limit is sufficient for the early diagnosis and treatment of cancers.

**FIGURE 4:**
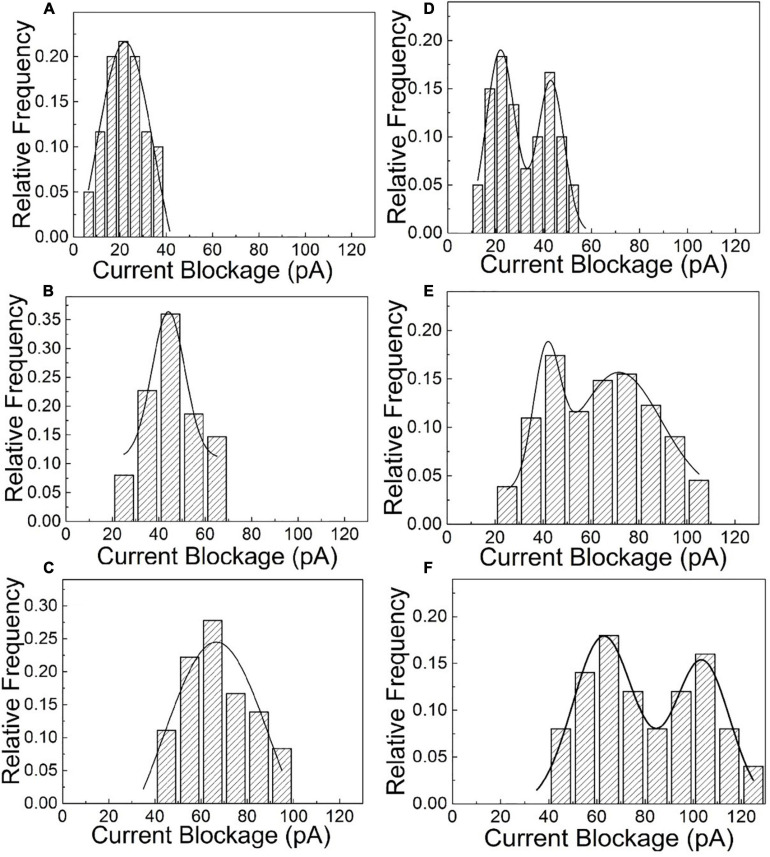
The statistical amplitude histograms of the blocked translocation events for wild-type and mutant targets as the function of biased voltages. **(A–C)** Are statistical graphs at 250, 500, and 700 mV for wild-type targets, respectively. **(D–F)** Are statistical graphs at 250, 500, and 700 mV for SNP targets, respectively.

After the quantitative determination of SNP targets, experimental factors such as time and voltage have also been evaluated to optimize the detection accuracy. The reaction dynamics of the SNP assay has been checked in our experiments, as shown in Figures 3C,D. The recognition response is rapid in the presence of SNP targets. The capture frequency is growing over time and saturated after 30 min. The capture frequency is much higher after adding SNP targets. The DNA-probed Au nanoparticles are stable in aqueous solution, and the self-assembled dimers are stabilized by the strong binding affinity of the complementary hybridization between the DNA-Au probe and SNP sequences. Likewise, the capture frequency of the SNP targets has been explored at different voltages in Figure 4. The results display that a single peak of the magnitude distribution for wild targets is not influenced at the varied voltages, but the peak value increases with voltage increasing, which is in line with Ohm’s law of nanopore devices. For SNP targets, the double peaks of the magnitude distribution always exhibit at different biased voltages, and the capture rates of monomer and dimer particles are all enhanced at high voltages. The Au nanoparticles with appreciable size can produce distinct ionic current signatures through nanopores with a high signal-to-noise ratio.

## Conclusion

Single-nucleotide polymorphisms are the most abundant genetic variation, which are responsible for genetic disease prevalent in a population. Therefore, the detection of the subtle single-nucleotide discrimination is important to the earlier diagnosis and treatment of critical illness. In our work, a sensitive SNP detection system has been established by silicon nitride nanopore platform integrated with DNA-probed gold nanoparticles. The readout of single-nucleotide discrimination in nanopore sensing has been converted into the amplified signals of varied nanoparticles with comparable volume to the used nanopore. The DNA probes absorbed on nanoparticles have high selectivity and sensitivity to the SNP targets. Hence, the SNP of the p53 gene mutation in gastric cancer has been detected in the femtomolar concentration. In the same way, the robust nanopore biosensing strategy can be adapted to detect broad DNA mutations from diagnostics to targeted therapy of cancers.

## Data Availability Statement

The original contributions presented in the study are included in the article/Supplementary Material, further inquiries can be directed to the corresponding author.

## Author Contributions

YY, GX, ZW, DM, and LL did the experiments and data processing. LZW, BB, and LXW were involved in the data analysis and manuscript writing. All authors equally contributed to the manuscript and participated in the results and discussion.

## Conflict of Interest

The authors declare that the research was conducted in the absence of any commercial or financial relationships that could be construed as a potential conflict of interest.
