# Supplementary figures and images for: Sensitive Detection of Single-Nucleotide Polymorphisms by Solid Nanopores Integrated With DNA Probed Nanoparticles
Source: Front Bioeng Biotechnol. 2021 Jun 30;9:690747. doi: 10.3389/fbioe.2021.690747 (PMC8279778; doi:10.3389/fbioe.2021.690747)

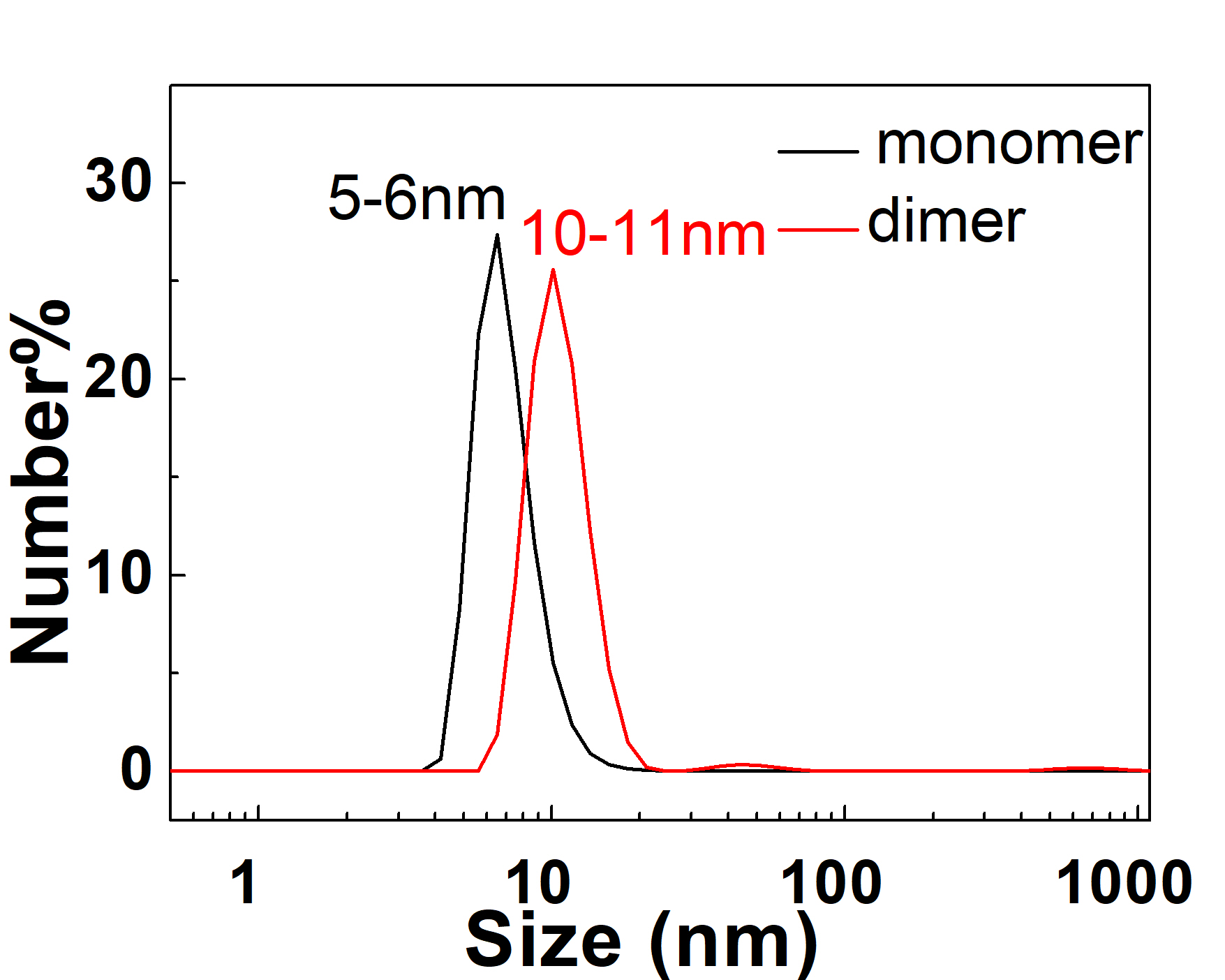

Supplement: Supplementary file 1 [file Image_1.JPEG]

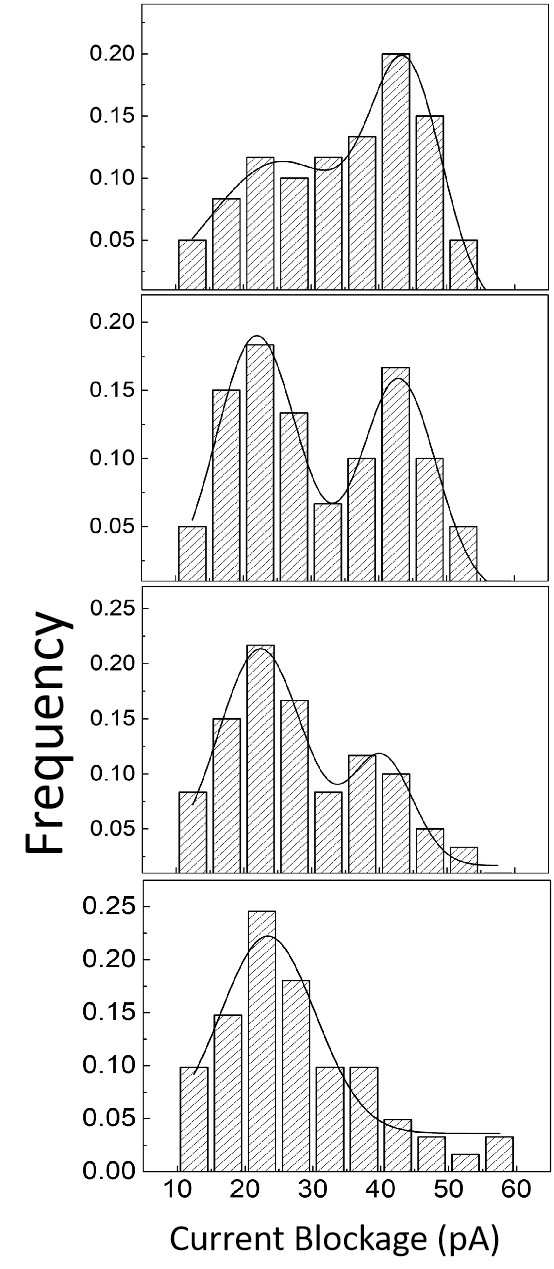

Supplement: Supplementary file 2 [file Image_2.JPEG]

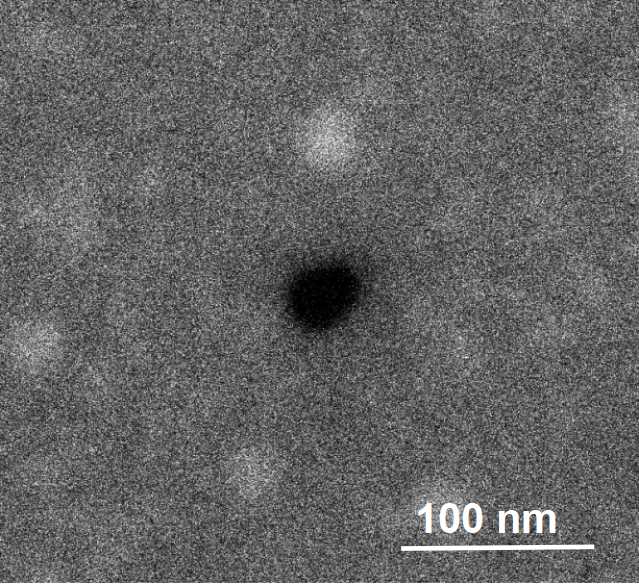

Supplement: Supplementary file 3 [file Image_3.JPEG]
